# rAAV-related therapy fully rescues myonuclear and myofilament function in X-linked myotubular myopathy

**DOI:** 10.1186/s40478-020-01048-8

**Published:** 2020-10-19

**Authors:** Jacob A. Ross, Hichem Tasfaout, Yotam Levy, Jennifer Morgan, Belinda S. Cowling, Jocelyn Laporte, Edmar Zanoteli, Norma B. Romero, Dawn A. Lowe, Heinz Jungbluth, Michael W. Lawlor, David L. Mack, Julien Ochala

**Affiliations:** 1 Centre of Human and Applied Physiological Sciences, School of Basic and Medical Biosciences, Faculty of Life Sciences & Medicine, King’s College London, London, UK; 2 British Heart Foundation Centre of Excellence, School of Cardiovascular Sciences, Faculty of Life Sciences & Medicine, King’s College London, London, UK; 3 Institut de Génétique Et de Biologie Moléculaire Et Cellulaire (IGBMC), INSERM U1258, CNRS UMR7104, Université de Strasbourg, Illkirch, France; 4 National Institute for Health Research, Great Ormond Street Institute of Child Health Biomedical Research Centre, University College London, 30 Guilford Street, London, UK; 5 Dubowitz Neuromuscular Centre, University College London, Great Ormond Street Institute of Child Health, 30 Guilford Street, London, UK; 6 Department of Neurology, Faculdade de Medicina (FMUSP), Universidade de São Paulo, São Paulo, Brazil; 7 Neuromuscular Morphology Unit, Myology Institute, Sorbonne Université, Centre de Référence de Pathologie Neuromusculaire Nord/Est/Ile-de-France (APHP), GH Pitié-Salpêtrière, Paris, France; 8 Division of Rehabilitation Science and Division of Physical Therapy, Department of Rehabilitation Medicine, University of Minnesota, Minneapolis, MN USA; 9 Randall Centre for Cell and Molecular Biophysics, School of Basic & Medical Biosciences, Faculty of Life Sciences & Medicine, Guy’s Campus, King’s College London, London, UK; 10 Department of Paediatric Neurology, Neuromuscular Service, Evelina’s Children Hospital, Guy’s and St Thomas’ Hospital National Health Service Foundation Trust, London, UK; 11 Department of Basic and Clinical Neuroscience, Institute of Psychiatry, Psychology and Neuroscience, King’s College London, London, UK; 12 Division of Paediatric Pathology, Department of Pathology and Laboratory Medicine and Neuroscience Research Center, Medical College of Wisconsin, Milwaukee, WI USA; 13 Department of Rehabilitation Medicine, University of Washington, Seattle, WA USA; 14 Institute for Stem Cell and Regenerative Medicine, School of Medicine, University of Washington, Seattle, USA; 15 Department of Biomedical Sciences, University of Copenhagen, Copenhagen N, Denmark

**Keywords:** Skeletal muscle, Congenital myopathy, Myotubularin, Myonuclear domain, Myofilament, Force production

## Abstract

X-linked myotubular myopathy (XLMTM) is a life-threatening skeletal muscle disease caused by mutations in the *MTM1* gene. XLMTM fibres display a population of nuclei mispositioned in the centre. In the present study, we aimed to explore whether positioning and overall distribution of nuclei affects cellular organization and contractile function, thereby contributing to muscle weakness in this disease. We also assessed whether gene therapy alters nuclear arrangement and function. We used tissue from human patients and animal models, including XLMTM dogs that had received increasing doses of recombinant AAV8 vector restoring *MTM1* expression (rAAV8-cMTM1). We then used single isolated muscle fibres to analyze nuclear organization and contractile function. In addition to the expected mislocalization of nuclei in the centre of muscle fibres, a novel form of nuclear mispositioning was observed: irregular spacing between those located at the fibre periphery, and an overall increased number of nuclei, leading to dramatically smaller and inconsistent myonuclear domains. Nuclear mislocalization was associated with decreases in global nuclear synthetic activity, contractile protein content and intrinsic myofilament force production. A contractile deficit originating at the myofilaments, rather than mechanical interference by centrally positioned nuclei, was supported by experiments in regenerated mouse muscle. Systemic administration of rAAV8-cMTM1 at doses higher than 2.5 × 10^13 ^vg kg^−1^ allowed a full rescue of all these cellular defects in XLMTM dogs. Altogether, these findings identify previously unrecognized pathological mechanisms in human and animal XLMTM, associated with myonuclear defects and contractile filament function. These defects can be reversed by gene therapy restoring *MTM1* expression in dogs with XLMTM.

## Introduction

The centronuclear myopathies (CNM) are a group of genetically and clinically heterogeneous early-onset muscle disorders, with phenotypes varying from severe cases with antenatal/neonatal onset and death in infancy if untreated, to mild cases with onset in adolescence or adulthood and a stable or only slowly progressive course. The severe X-linked form of CNM (also termed X-linked myotubular myopathy or XLMTM, OMIM 310,400) is caused by hemizygous mutations in the *MTM1* gene (encoding the myotubularin protein) and affects approximately 1:50,000 males [17–19, 41]. The precise pathogenesis of XLMTM remains uncertain, although several observations, including altered excitation–contraction coupling, reduced muscle fibre size, neuromuscular junction (NMJ) abnormalities and alterations to autophagy have been proposed as contributing mechanisms [17, 18]. A particular interesting unresolved question concerns the relationship between mislocalization of nuclei and the often profound weakness observed in this condition. Hence, the overall aim of the present work is to gain deeper insights into the underlying mechanisms of XLMTM.

Skeletal muscle fibres are large cells filled with contractile filaments, and contain many nuclei (often termed myonuclei) [30]. During development, nascent fibres possess myonuclei that are in the centre or core of the fibre, buried among myofibrils; these then translocate to the periphery where they remain throughout maturity. Similarly, during muscle regeneration, nascent or partially repaired fibres possess internal nuclei, which then translocate to the periphery [30]. Myonuclei are then regularly distributed throughout the fibre, allowing the control of gene transcription for a defined volume of cytoplasm termed the myonuclear domain (MND). The maintenance of an optimal MND size is essential for the intrinsic force-generating capacity of myofibres and overall cellular function [22, 28].

In XLMTM, two populations of myonuclei exist: those that are positioned at the periphery of muscle fibres, and those that are abnormally positioned internally, at or near the centre of the fibres [17, 18]. Given the importance of nuclear positioning for muscle function, we hypothesized that mislocalization of nuclei might be a critical pathogenic factor in XLMTM. Although the presence of central nuclei in XLMTM is well documented [19], to our knowledge, myonuclear domain sizes, variability and overall distribution of peripheral and central nuclear populations have not been fully explored in the context of this disease. However, these aspects so far neglected in XLMTM-related research may be of particular importance to the pathogenesis of this severe condition, considering that altered nuclear distribution may then be associated with aberrant global gene transcription (or synthetic activity), contractile protein content and overall muscle fibre dysfunction. In the current study, we first aimed to verify that hypothesis by using muscle tissue from humans with *MTM1* mutations and animal models of XLMTM, including the *Mtm1* knockout (KO) mouse, and the well-established canine colony with a mutation in *MTM1* [1, 5–7, 9, 13, 23, 38]. We also sought to investigate whether the presence of centrally located nuclei might directly interfere with muscle contraction, since these nuclei are buried between the contractile filaments. We isolated membrane-permeabilized muscle fibres and ran a series of functional and morphological measures including an evaluation of the 3D spatial arrangement of myonuclei in relation to myofibre size, using our image analysis algorithm applied to confocal images [22, 31]. We then correlated these results with a marker of global nuclear transcriptional output, muscle architecture at the ultrastructural level, and measurements of intrinsic contractile force generation.

The XLMTM canine model harbouring a p.N155K mutation in the *MTM1* gene is similar to human patients in its capacity to produce significant pathology and weakness, although in dogs, the clinical course is different, showing disease progression after an initial period of normal development, as attested by a rapidly increasing inability to stand, walk, and feed [2, 6]. A previous study administered a single dose of recombinant AAV8 vector (rAAV8) expressing the wild type canine *MTM1* cDNA (rAAV8-cMTM1) to 10 week old dogs with XLMTM, at three doses: 5.0 × 10^12^ (AAVLow), 2.5 × 10^13^ (AAVMid), and 8.0 × 10^13^ (AAVHigh) vg.kg^−1^. Whilst the AAVLow doses had a minimal effect in these dogs, AAVHigh and AAVMid were able to restore excitation–contraction coupling, increase myofibre size and muscle volume by ~ 50%, rescue limb strength to near normal levels, and establish normal gait and survival rate [6, 9, 23]. This previous canine gene therapy study provided a unique opportunity to test whether administration of rAAV-cMTM1 had beneficial effects on myonuclear localization, MND volumes and muscle fibre force production specifically at the level of the contractile machinery. We also ran similar tests on muscle fibres from patients with XLMTM and healthy control subjects, and found similarities in these disease hallmarks between humans, mice and dogs.

## Materials and methods

### Human subjects

All tissue was consented, stored and used in accordance with the Human Tissue Act, UK, under local ethical approval (REC 13/NE/0373). Details of patients and controls are given in Table 1.

**Table 1:** Patient and control muscle biopsy samples used

Age	Gender	Gene mutation	Age of onset (severity)	Source
4 years	Male	*MTM1 c.339 T* > *A*	Infancy (severe)	Sao Paulo, Brazil
18 years	Female	*MTM1 c.1061del*	Infancy (symptomatic carrier)	Paris, France
2 months	Male	*MTM1 c.523A* > *G*	Birth (severe)	Paris, France
25 years	Female	–	–	London, UK
20 years	Female	–	–	Sao Paulo, Brazil
16 years	Male	–	–	Sao Paulo, Brazil
20 years	Male	–	–	London, UK

### Canine model

The XLMTM Labrador/Beagles used in the present experiments were part of a previous gene therapy study [7, 23]. Briefly, dogs were handled according to principles outlined in the National Institutes of Health (NIH) *Guide for the Care and Use of Laboratory Animals*. XLMTM-affected dogs carried a p.N155K mutation in the *MTM1* gene, and were bred in a colony maintained at the University of Washington. Twelve of these XLMTM dogs were injected systemically with a saline solution (XLMTM, N = 3) or with three different doses of a rAAV8 vector expressing the wild type canine *MTM1* cDNA (rAAV8-cMTM1): 5.0 × 10^12^ (AAVLow, N = 3), 2.5 × 10^13^ (AAVMid, N = 3), and 8.0 × 10^13^ (AAVHigh, N = 3) vg.kg^−1^ at the age of 10 weeks. All animals, including wild-type control dogs (N = 3) were sacrificed between 39 and 41 weeks of age (end of the study) or when reaching humane euthanasia criteria (pentobarbital anaesthetics overdose). Skeletal muscle specimens were then obtained from biceps femoris.

### Mouse model

Six-week old homozygous *MTM1*-deficient (Mtm1 KO, N = 3) and age-matched wild-type (WT, N = 3) littermates were used in the present study [1, 5, 38]. Mice were euthanized by cervical dislocation and tibialis anterior muscles were dissected. Care and manipulation of mice were performed in accordance with national and European legislations on animal experimentation (Com’eth N°01,594.02).

### Muscle injury model

Mice were bred and experimental procedures were carried out in the Biological Services Unit, University College London Great Ormond Street Institute of Child Health, in accordance with the Animals (Scientific Procedures) Act 1986. Experiments were performed under Home Office license numbers 70/ 8389. Experiments were approved by the local University College London Ethics Committee before the license being granted. The induction of muscle degeneration using notexin was carried out as described previously [14]. Notexin is a myotoxin from snake venom, which results in necrosis of muscle fibres. Following injury, muscles are able to fully regenerate. Briefly, eight-week old wild-type mice (N = 4) were anaesthetized under isoflurane. An intramuscular injection of notexin (10 µl of a 10 µg/ml solution) was administered into the tibialis anterior muscle of the right leg using a Hamilton syringe. The left leg served as a contralateral control. Following recovery, mice were left for eight weeks to allow full muscle regeneration. Following this, tibialis anterior muscles from both legs were harvested, with half the muscle used for skinned muscle fibre preparation (see below), and half cryo-embedded for cryo-sectioning and histology (as described previously in [32]).

## Solutions

Relaxing and activating solutions contained 4 mM Mg-ATP, 1 mM free Mg^2+^, 20 mM imidazole, 7 mM EGTA, 14.5 mM creatine phosphate, and KCl to adjust the ionic strength to 180 mM and pH to 7.0*.* The concentrations of free Ca^2+^ were 10^–9.00^ M (relaxing solution) and 10^–4.50^ M (activating solution).

### Muscle fibre permeabilization

In the current study, because of the low number of muscle fibres expressing the type IIa and IIx myosin heavy chains that we extracted from humans and canines, we focused our attention on slow/type I fibres. Mouse tibialis anterior muscles are known to be exclusively composed of type II isoforms, and hence all mouse data is of this category. Muscle samples were placed in relaxing solution at 4 °C. Bundles of approximately 50 myofibres were dissected and treated with skinning solution (relaxing solution containing glycerol; 50:50 v/v) for 24 h at 4 °C, after which they were transferred to − 20 °C.

### Single myofibre force production

**Fig. 1:**
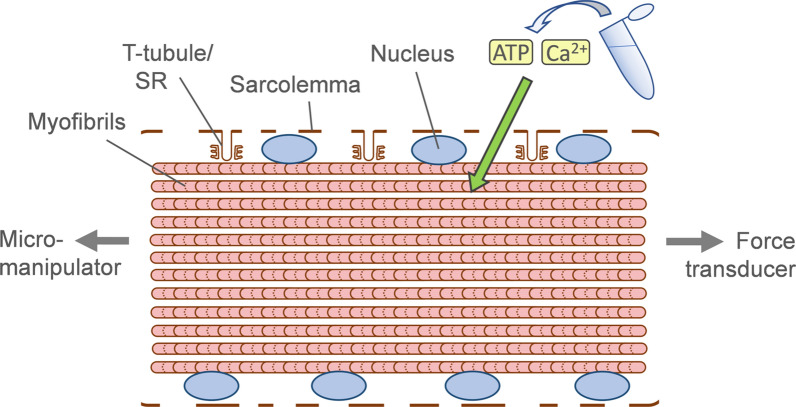
Skinning/permeabilization of single muscle fibres and measurement of contractile force. Muscle fibres are chemically membrane-permeabilized with a glycerol-containing relaxing solution, to create ‘holes’ in the cell membrane (sarcolemma) [8]. Fibres are then either (**a**) fluorescently immunolabelled, or (**b**) assessed for contractile force generation attached via a force transducer. In the latter case, contraction is directly triggered by the addition of Ca^2+^ and ATP (facilitated by membrane permeabilization). Thus, upstream events that normally contribute to muscle contraction are bypassed (e.g. electrical stimulation or excitation–contraction coupling). Note that these skinned single muscle fibres retain all their peripheral and central nuclei (see also Figs. 2a, 3a, 4a)

On the day of experiment, single myofibres were dissected. They were then individually attached between connectors leading to a force transducer and a lever arm system (model 1400A; Aurora Scientific). Sarcomere length was set to ≈2.50 µm and the temperature to 15 °C [22]. Fibre cross-sectional area (CSA) was estimated from the width and depth, assuming an elliptical circumference. The absolute maximal isometric force generation was calculated as the difference between the total tension in the activating solution (pCa 4.50) and the resting tension measured in the same myofibre while in the relaxing solution (pCa 9.0). Specific force was defined as absolute force divided by CSA. Figure 1 shows a schematic of muscle fibre permeabilization and activation of contraction for measurement of force production.

### Nuclear organization of single fibres

Single muscle fibres were dissected following the same procedure as above. Arrays of approximately nine myofibres were prepared at room temperature (RT). For each myofibre, both ends were clamped to half-split copper meshes designed for electron microscopy (SPI G100 2010C-XA, width, 3 mm), which had been glued to cover slips (Menzel-Gläser, 22 × 50 mm, thickness 0.13–0.16 mm). For the measurement of nuclear coordinates, fibres were mounted at a fixed sarcomere length of ≈2.20 µm. This was a prerequisite for exact determination of nuclear spatial organization as it allowed accurate comparisons between myofibres [22, 31–33].

At RT, arrays were fixed in 4% PFA for 10 min, washed × 3 in phosphate buffered saline (PBS), further permeabilized in 0.1% triton-X100/PBS for 10 min, and subsequently subjected to actin staining (Rhodamine-conjugated Phalloidin at 1:100 in PBS, Molecular Probes, R415) and nuclear staining (DAPI at 1:1000 in PBS, Molecular Probes, D3571). Images were acquired using a confocal microscope (Zeiss Axiovert 200, objectives 20×, 40 × and 100 × ) equipped with CARV II confocal imager (BD Bioscience). To visualize muscle fibres in 3D, stacks of 100 images were acquired (1 µm *z* increments). Membrane-permeabilized fibres did not display any Pax7 positive satellite cells. In order to measure how ordered the nuclear distribution for a particular fibre was, the centroids of nuclei were identified in the 3D Z-stacks, along with the fibre boundaries, and analysed with a custom-made MATLAB script [22]. Calculations of MND size and order scores were based on algorithms by Bruusgaard and co-workers [4]. For order score, a theoretical optimal and a theoretical random distribution was simulated, based on fibre dimensions and nuclear number. We denote the experimental, random and optimal means by M_E_, M_R_ and M_O_, respectively. An 'order-score', “g”, was then calculated as: g = (M_E_-M_R_)/(M_O_-M_R_).

### Immunofluorescent labelling

*Immunofluorescent labelling of single fibres*: fibres were PFA-fixed and Triton-permeabilized as described above (“nuclear organization of single fibres”). Fibres were blocked in 10% goat serum in PBS (Sigma Aldrich, G9023) for 30 min, and treated with either acetyl-Histone H3 (Lys9/Lys14) (Cell Signalling, #9677) or myosin heavy chain slow/type I (Santa Cruz, A4.951). Secondary antibodies were labelled with Alexa Fluor® 594 or 488, respectively (Invitrogen), diluted in goat serum blocking buffer. After washing, slides were mounted in Fluoromount (Southern Biotech). Myosin heavy chain identification was carried out on all the fibres that were functionally or morphologically tested. As the majority of the myofibres expressed the slow/type I myosin heavy chain, we focused on slow fibres only in the present study.

*Immunohistochemistry on cryosections*: myosin heavy chain isoform detection was carried out on 10 µm transverse cryosections of mouse tibialis anterior muscles, as described previously [32]. Extracellular matrix was stained with Alexa647-conjugated wheat germ agglutinin (WGA) to demarcate muscle fibre boundaries.

### Electron microscopy

Muscle tissue was fixed in 2.5% glutaraldehyde, processed at the Medical College of Wisconsin electron microscopy (EM) Core Facility. Percentage of total fibre area occupied by myofibrils was determined by colouring each myofibril area in a cross-sectional image black and using ImageJ software (v.1.36b; National Institutes of Health, Bethesda, MD) to threshold the scanned image and measure the percentage of black area in sections (at least five images per animal) [26, 27].

### LC–MS/MS identification and quantitative analysis of protein

Preparation: 7-mm long muscle fibres were dissected and their CSA calculated as above. These fibres were then placed in tubes containing 25 µl Tris-Triton lysis buffer (10 mM Tris (pH 7.4), 100 mM NaCl, 1 mM EDTA, 1 mM EGTA, 1% Triton X-100, 10% Glycerol, 0.1% SDS, 0.5% Deoxycholate). Prior to enzymatic digestion and labelling, the samples were loaded into a stack gel for lysis buffer clean up to eliminate chemical interference at the labelling stage and to compress the whole proteome into a single band. Sample volumes were dried by half in a SpeedVac (Thermo Fisher Scientific) with the volume replaced by Laemmli buffer (2x) and heated for 10 min at 96 °C. Reduced samples were loaded onto a 10% BisTris NuPAGE gel and resolved for 10 min (100 V; 59 mA; 6 watts) to ‘stack’ the whole sample into a single band. Protein bands were visualized using Imperial protein stain (Thermo Fisher Scientific).

Digestion and peptide labelling with TMT: In-gel reduction, alkylation and digestion with trypsin were performed on all the samples prior to subsequent isobaric mass tag labelling [35]. Each sample was treated individually with labels added at a 1:1 ratio.

LC–MS/MS tandem mass spectrometry: The combined TMT labelled peptide samples were resuspended in a solution containing water:acetonitrile:trifluoroacetic acid (98%:2%:0.05%) and analysed by LC–MS/MS. Chromatographic separations were performed using an Ultimate 3000 UHPLC system (ThermoFisherScientific, UK). A 10 µl injection of peptides was resolved by reversed phase chromatography on a 75 µm C18 column (50 cm) using a three step linear gradient of acetonitrile in 0.1% formic acid. The gradient was delivered to elute the peptides at a flow rate of 250 nL/min over 120 min. The eluate was ionized by electrospray ionization using an Orbitrap Fusion Lumos (ThermoFisherScientific, UK) operating under Xcalibur v4.1. The instrument was programmed to acquire in automated data-dependent switching mode, selecting precursor ions based on their intensity for sequencing by Higher-energy C-trap dissociation (HCD) for peptide identification and reporter ion fragmentation. Selection of precursor ions based on their intensity for sequencing by HCD in a TopN method. The MS/MS analyses were conducted using higher than normal collision energy profiles that were chosen based on the mass-to-charge ratio (m/z) and the charge state of the peptide. To increase fragmented peptide coverage and reporter ion intensities, a further Synchronous Precursor Scan (SPS) of the Top 5 most intense peaks using MS3 was performed.

Database Searching: Raw mass spectrometry data were processed into peak list files using Proteome Discoverer (Thermo Scientific; v2.2). The raw data file was processed and searched using the Mascot search algorithm (v2.6.0; www.matrixscience.com) and the Sequest search algorithm [10] against the current Mouse database curated within Uniprot.

Bioinformatics: Following processing with Proteome Discoverer, the result file was exported into Perseus (v1.6.3; https://www.perseus-framework.org) for qualitative and quantitative data analysis.

### Statistical analysis

Data are presented as mean ± SEM. The statistical analysis was performed using SPSS Statistics 23 software (IBM) and included normality tests, as well as t-tests, ANOVAs and Pearson product moment correlation (to evaluate linear relationships). Statistical significance was set to *P* < 0.05 (*), *P* < 0.01 (**) and *P* < 0.001 (***).

## Results

### Human XLMTM muscle fibres display myonuclear perturbations

**Fig. 2:**
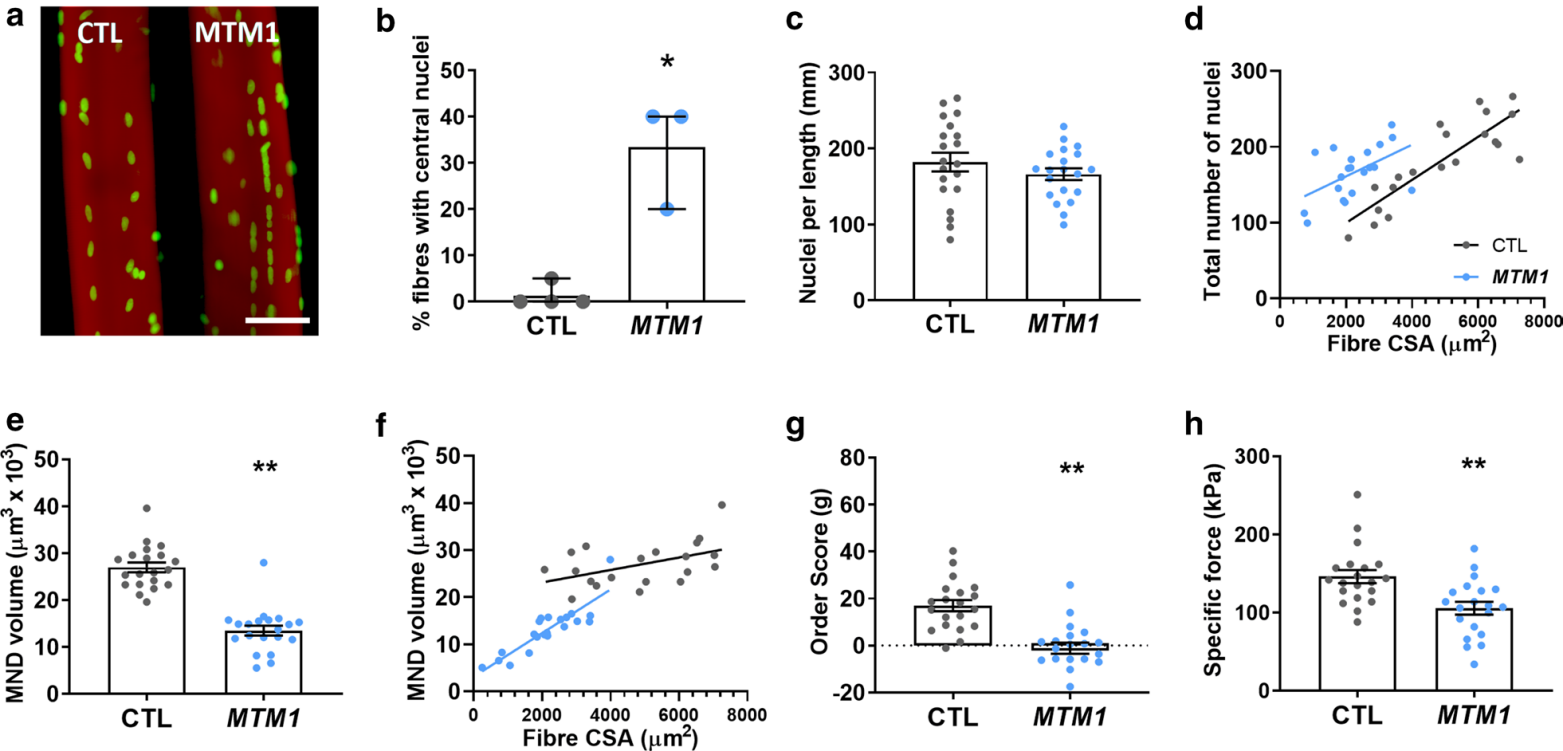
Muscle fibres from XLMTM patients have increased density and irregular spacing of myonuclei, and reduced force-generating capacity. Data from slow/type I muscle fibres from XLMTM patients (MTM1, N = 3) and healthy controls (CTL, N = 4) (n = total of 20 fibres per group). **a** Representative single muscle fibres from each group, stained for actin (rhodamine phalloidin; red) and nuclei (DAPI; green). The example XLMTM fibre is from the first patient in Table 1. Scale bar: 50 μm. **b** Proportion of muscle fibres containing internal or central nuclei. Each data point corresponds to the mean per subject. **c** Number of myonuclei per individual myofibre. **d** Myonuclear number was linearly related to fibre CSA for all the groups (*P* < 0.05). **e** Myonuclear domain volume per individual muscle fibre. **f** Myonuclear domain size was linearly related to fibre CSA for all the groups (*P* < 0.05). **g **Order score (g), an algorithm to assess the regularity of nuclear spacing; a lower score indicates more irregular spacing and more nuclear clustering. **h** Specific force defined as absolute (maximal) force divided by CSA. Individual data points for **c–h** correspond to individual muscle fibres. Column graphs show mean ± S.D. Scatter graphs show linear regression lines. Asterisks denote a significant difference compared with CTL (*P < 0.05; **P < 0.01; t-test)

We isolated single muscle fibres from patients with XLMTM (MTM1, N = 3 patients, n = 20 muscle fibres total) and healthy control donors (CTL, N = 4 patients, n = 20 fibres total). Due to the low proportion of type II myosin heavy chain isoforms in these muscle samples, only fibres positive for type I myosin were analyzed. We then analyzed myonuclear organization using 3D confocal microscope reconstructions (Fig. 2a). As expected, XLMTM patients had a large proportion of fibres with centralized nuclei (assessed using individual confocal Z-slices throughout the fibre, Fig. 2a, b). In addition, mean fibre cross sectional area (CSA) was smaller in patients than in healthy controls (compare distributions of CSA in Fig. 2d, f). The total number of myonuclei per mm fibre length was not significantly different between groups (Fig. 2c). However, as reported previously [22, 31–33], this parameter was positively and linearly correlated with the CSA, with larger fibres possessing more nuclei (Fig. 2d). Hence, for any given CSA, the number of nuclei per mm fibre length was greater in XLMTM patients than in healthy controls. This demonstrates an abnormally high density of myonuclei in XLMTM fibres. In agreement with this, myonuclear domain (MND) sizes were dramatically smaller in patients compared to controls (Fig. 2e). As for the previous parameter of number of myonuclei per mm fibre length, MND values were positively and linearly correlated with CSA in both groups (Fig. 2f). However, for any given CSA, MND was smaller in patients than controls (Fig. 2f). Although fibres with lower CSAs are known to have smaller MNDs (hence the positive linear relationship in Fig. 2f), the regression lines and data points would suggest that even though XLMTM fibres are small, they have unusually reduced MND sizes compared to healthy controls, suggesting a primary effect of disease, rather than a secondary effect of hypotrophy.

MND measurement provides valuable information on the average volume of cytoplasm controlled by each myonucleus; however, it does not take into account variability between individual domains, nor the overall spatial arrangement/organization of myonuclei. To investigate this, we calculated a distribution or order score (‘g’) as described previously [4, 22, 31–33]. g was significantly lower (i.e. myonuclei are more disorganized in their overall distribution) in XLMTM compared to healthy control fibres (Fig. 2g).

### Human XLMTM fibres exhibit a disrupted force generating capacity originating at the myofilaments

Optimal MND sizes and nuclear positioning are essential for cellular and contractile function [22, 28]. To measure the force production of myofibres at the contractile level, we measured the absolute steady-state isometric force at *saturating* [Ca^2+^] (pCa 4.50) of membrane-permeabilized fibres (20 fibres per group). In this system, externally applied ATP and Ca^2+^ directly activate contraction at the level of the myofilaments. Thus, upstream events that normally contribute to muscle contraction are bypassed (e.g. electrical stimulation or excitation–contraction coupling), and the properties of myofilament force production are examined in isolation (see Fig. 1 for schematic diagram). In correlation with the nuclear abnormalities, specific force (defined as absolute (maximal) force divided by CSA) was lower in XLMTM patient fibres compared to healthy controls (Fig. 2h).

### The *Mtm1* knockout (KO) mouse model of XLMTM displays myonuclear perturbations and disrupted force generating capacity

**Fig. 3:**
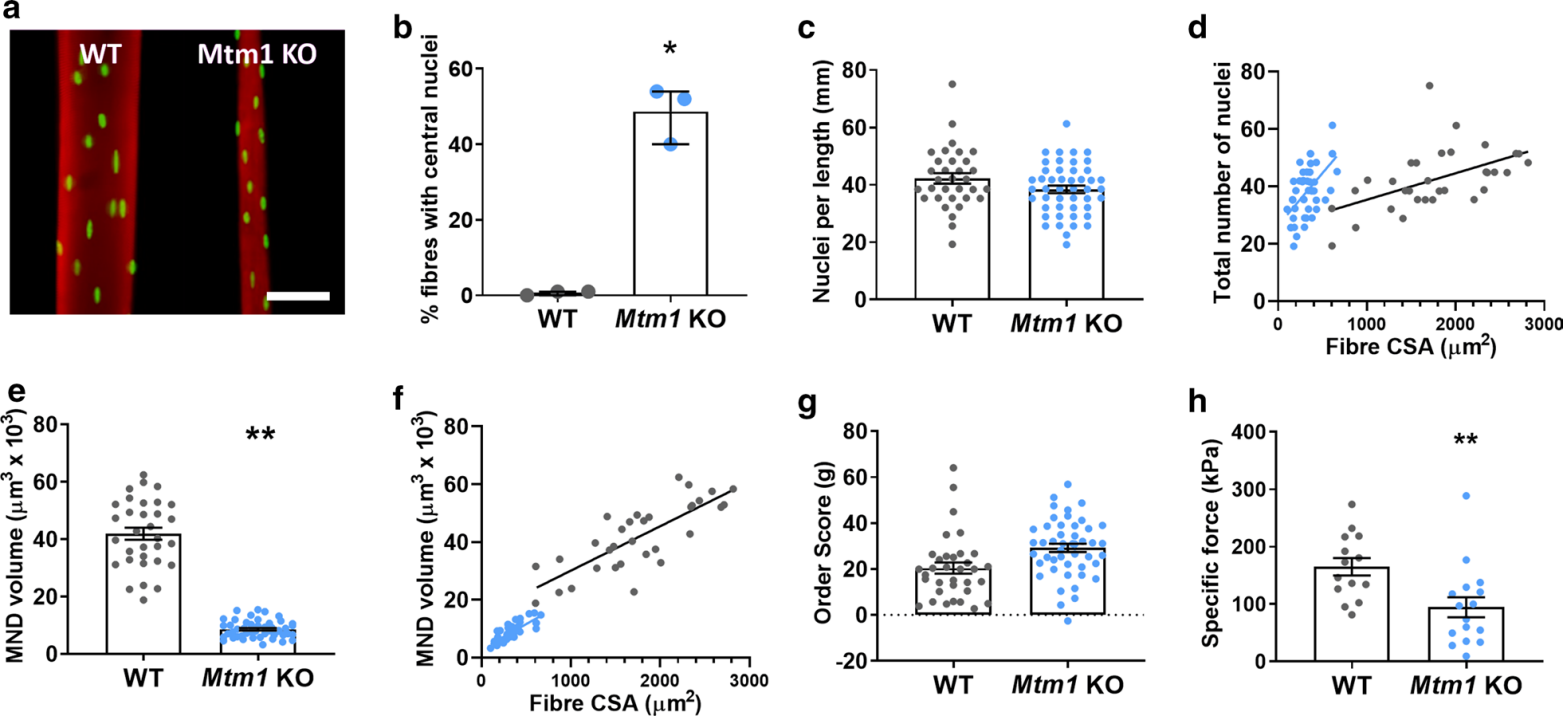
Muscle fibres from *Mtm1* knockout (KO) mice have increased density of myonuclei, and reduced force-generating capacity. Data obtained from healthy control mice (WT, N = 3 animals, n = total of 46 muscle fibres) and *Mtm1* KO animals (N = 3 animals, n = total of 63 fibres). **a** Typical single myofibres from each group, stained for actin (rhodamine phalloidin; red) and nuclei (DAPI; green). Scale bar: 50 μm. **b** Proportion of muscle fibres containing internal or central nuclei. Each data point corresponds to the mean per animal. **c** Number of myonuclei per individual myofibre. **d** Myonuclear number was linearly related to fibre CSA for all the groups (*P* < 0.05). The regression lines were significantly more elevated for Mtm1 KO than WT (*P* < 0.05). **e** Myonuclear domain volume per individual muscle fibre. **f** Myonuclear domain size was linearly related to fibre CSA for all the groups (*P* < 0.05). **g** Order score (g). **h** Specific force defined as absolute (maximal) force divided by CSA. Individual data points for **c–h** correspond to individual muscle fibres. Column graphs show mean ± S.D. Scatter graphs show linear regression lines. Asterisks denote a significant difference compared with CTL (*P < 0.05; **P < 0.01; t-test)

To investigate whether similar disruptions occur in muscle fibres expressing other myosin isoforms, we used a well-known mouse model of the disease, the *Mtm1* KO mouse, where skeletal muscles are mainly composed of the fast type IIx and IIb myosin heavy chains [1]. We then tested 109 muscle fibres from 6 mice: 3 healthy wild types (WT, n = 46 fibres total) and 3 *Mtm1* KO mice (n = 63 fibres total). Consistent with our human results, muscle fibre CSA was significantly reduced, and central nucleation significantly increased, in *Mtm1* KO mice compared to controls (Fig. 3a, b, d). Also in agreement with our human data, *Mtm1* KO mice exhibited increased numbers of myonuclei (Fig. 3d) and smaller MND volumes than WT (Fig. 3e, f). However, unlike XLMTM patients, order score (g) was unchanged in *Mtm1* KO mice, suggesting no alterations in the regularity of spacing between myonuclei (Fig. 3g). The nuclear changes described above were associated with a low force generating capacity originating specifically at the level of the myofilaments (Fig. 3h), in agreement with the human data.

Altogether, our findings suggest a novel etiology in XLMTM, associated with MND changes, altered nuclear arrangement and reduced force production related to the myofibrils themselves.

### Delivery of wild type *MTM1* gene rescues myonuclear density and distribution defects in dogs with XLMTM

**Fig. 4:**
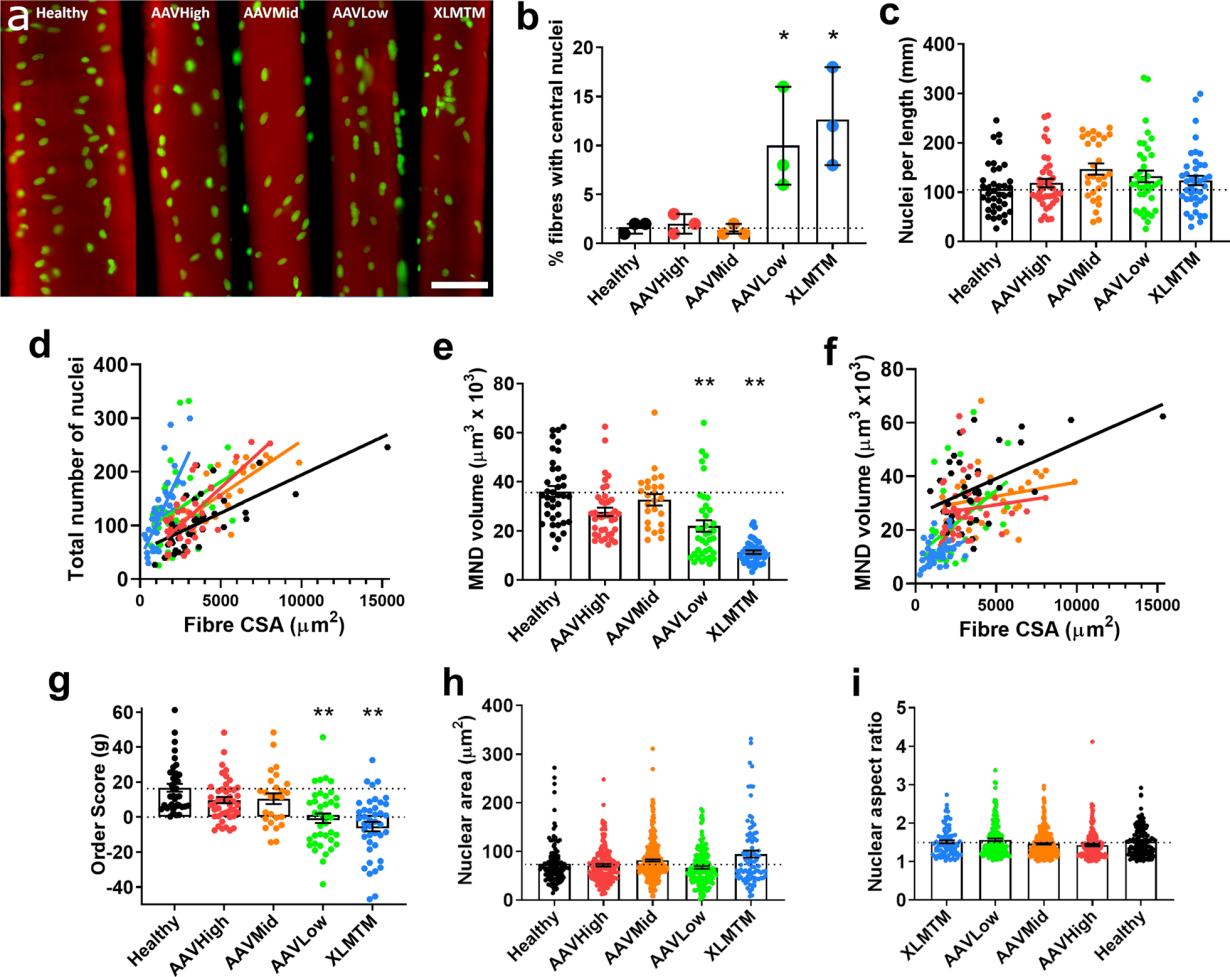
Delivery of wild type *MTM1* gene rescues myonuclear density and distribution defects in dogs with XLMTM. Dogs from a previous study were separated into the following groups (N = 3 per group): healthy controls, injected with saline; dogs expressing the p.N155K mutation in the *MTM1* gene, injected with a saline solution (XLMTM group); and dogs expressing the *MTM1* mutation but given 3 different doses of rAAV8-cMTM1 (AAVLow, AAVMid, AAVHigh). **a** Typical single myofibres from each group, stained for actin (rhodamine phalloidin; red) and nuclei (DAPI; green). Scale bar: 50 μm. **b** Proportion of muscle fibres containing internal or central nuclei. Each data point corresponds to the mean per animal. **c** Number of myonuclei per individual myofibre. **d** Myonuclei number was linearly related to fibre CSA for all the groups (*P* < 0.05). The regression lines were significantly more elevated for XLMTM and AAVLow dogs versus all other groups (*P* < 0.05). **e** Myonuclear domain volume per individual muscle fibres. **f** Myonuclear domain size was linearly related to fibre CSA for all the groups (*P* < 0.05). **g** Order score (g). Graphs showing shape quantifications for nuclei: projected area in the 2D (X–Y) plane, **h** and aspect ratio **i**. For **c**–**g**, data points correspond to individual muscle fibres. For **h** and **i**, individual data points correspond to individual nuclei. Column graphs show mean ± S.D. Scatter graphs show linear regression lines. Asterisks denote a significant difference compared with CTL (*P < 0.05; **P < 0.01; one-way ANOVA)

We isolated 186 individual myofibres (minimum of 10 fibres per dog) from 15 dogs: 3 healthy control dogs (Healthy), 3 animals expressing the p.N155K mutation in the *MTM1* gene which were injected with a saline solution (XLMTM), and 9 dogs also carrying the mutation but where three different doses of rAAV8-cMTM1 were administered (AAVLow, N = 3; AAVMid, N = 3; AAVHigh, N = 3). As expected from previous published data [6, 9, 23], a high proportion of muscle fibres from XLMTM and AAVLow groups possessed central nuclei, but in AAVMid and AAVHigh groups, this was rescued to levels indistinguishable from healthy controls (Fig. 4b). In addition, mean fibre CSA was smaller in XLMTM and AAVLow dogs, but approaching that of the healthy control group following AAVMid and AAVHigh treatments (compare distributions of CSA in Figs. 4d, f). XLMTM and AAVLow dogs exhibited increased numbers of myonuclei (Fig. 4d), smaller MND volumes (Fig. 4e, f), and reduced order score (g), suggesting a higher density of myonuclei, and irregularities in their spacing thoughout the fibre (Fig. 4g). This is in agreement with the findings in XLMTM patients. Treatment with AAVMid and AAVHigh doses was capable of restoring all these parameters to levels that were comparable with healthy controls (Fig. 4a–g).

Myonuclear size scaling is known to happen in response to changes in nuclear number and MND volumes in order to modulate synthetic activity [42]. Here, even though we found higher numbers of nuclei and smaller MND sizes in XLMTM and AAVLow animals, we did not observe any difference in myonuclear size (projected area in the 2D X–Y plane) or aspect ratio between groups (Fig. 4h, i).

These findings partially confirm our initial hypothesis and imply that there is an increased number of myonuclei within myofibres of the canine model of XLMTM, which results in smaller MND sizes. Myonuclei are arranged in both the centre and periphery of myofibres in diseased animals and are inconsistent or more unevenly spaced (i.e. lower order (‘g’) score). AAVMid and AAVHigh doses of the canine *MTM1* gene were able to fully restore myofibre CSAs, and fully rescue MND sizes and nuclear distribution parameters to those of healthy animals (Fig. 4d–g).

### Delivery of wild type *MTM1* gene rescues disrupted histone acetylation in XLMTM dogs

**Fig. 5:**
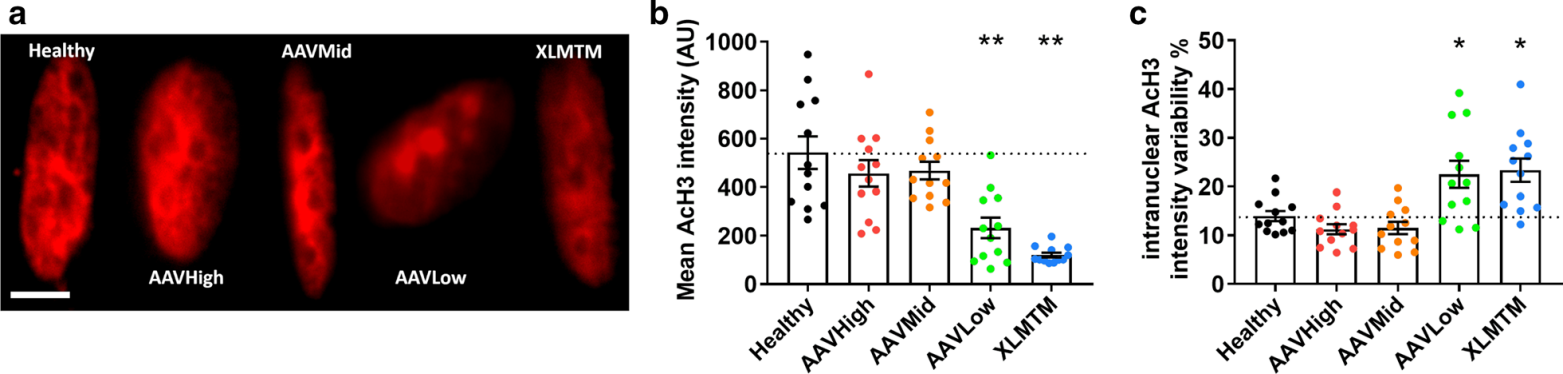
Delivery of wild type *MTM1* gene rescues disrupted histone acetylation in XLMTM dogs. acetyl histone H3 (Lys9/Lys14) (referred to her as AcH3) is a histone modification marking actively transcribed regions of DNA. Mean fluorescence intensity of immunolabelled AcH3 correlates with global gene transcription levels [22, 31]. Dogs from 5 groups were analysed (N = 3 per group, see legend from Fig. 2): Healthy, XLMTM, AAVLow, AAVMid, and AAVHigh. **a** Representative single myonuclei from each group, stained for AcH3 (red). Scale bar: 10 μm. **b** Mean AcH3 intensity calculated for 10 nuclei per fibre. **c** Mean standard deviation of pixel intensity, as a percentage of mean pixel intensity, of AcH3 staining within individual nuclei. This is a measure homogeneity of immunolabelling within each nucleus, and suggests more irregular distribution of chromatin and/or active regions of DNA in XLMTM and AAVLow myonuclei. Data points correspond to individual muscle fibres (10 nuclei per fibre, from a total of 12 fibres per treatment group). Column graphs show mean ± S.D. Asterisks denote a significant difference compared with CTL (* *P* < 0.05; ** *P* < 0.01; one-way ANOVA)

The above nuclear changes might be associated with global gene transcription abnormalities, since nuclear cooperation is known to be affected by nuclear spacing within muscle fibres [37]. To investigate this specific point, we used immunofluorescence to label acetyl histone H3 (Lys9/Lys14) (AcH3). This histone modification is well documented as a marker of actively transcribed regions of DNA, and nuclear abundance/fluorescence intensity of this marker is positively correlated with global nuclear transcription levels [22, 31]. The mean fluorescence intensity of AcH3 within individual myonuclei was significantly lower in XLMTM and AAVLow dogs than in healthy, AAVMid and AAVHigh animals (Fig. 5a, b). Next, we analysed the variability of pixel intensities within each single nucleus (standard deviation of all AcH3 positive pixel values, as a percentage of the mean value). We observed that the AcH3 intensity variability within each nucleus was significantly greater in XLMTM and AAVLow animals than in the other groups (Fig. 5c), suggesting that the distribution of this marker is altered within each nucleus, likely corresponding to altered chromatin organization and/or altered distribution of active domains of DNA. Together with the reduced mean fluorescence intensity of AcH3, this suggests that XLMTM and AAVLow animals display reduced or altered global transcriptional output.

### Delivery of wild type *MTM1* gene rescues contractile protein content and force generation in XLMTM dogs

**Fig. 6:**
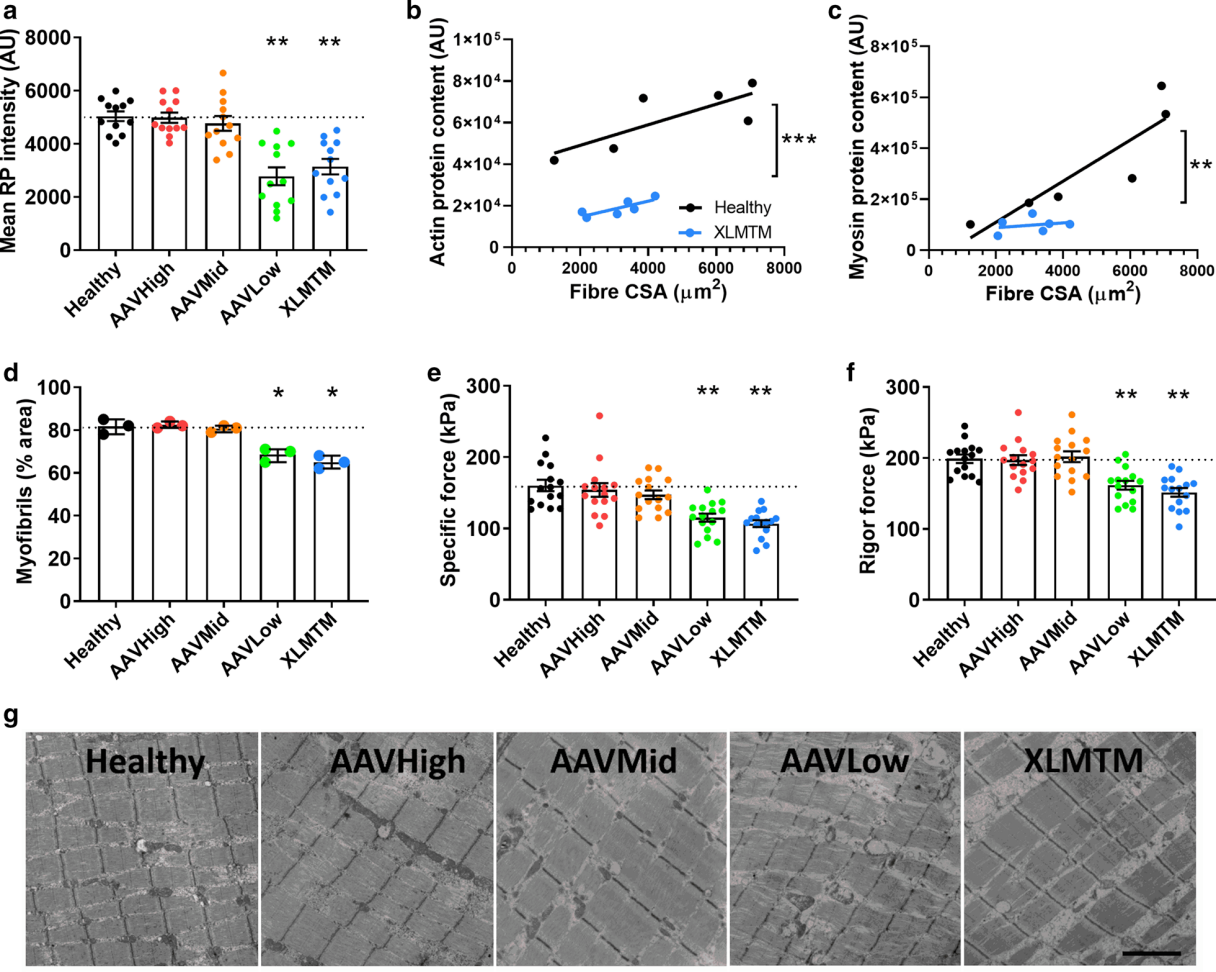
Delivery of wild type *MTM1* gene rescues contractile protein content and force production in XLMTM dogs. Dogs from 5 groups were analysed (N = 3 per group, see legend from Fig. 2). **a** Mean fluorescence intensity of rhodamine phalloidin (RP) calculated for individual fibres, as a measure of actin density/content. Total myosin **(b)** and actin **(c)** protein content in fibres from XLMTM and Healthy dogs, as assessed by proteomics (n = 6 fibres per group). **d** Myofilament content, as assessed on transmission electron micrographs of longitudinal muscle sections, measured as proportion of the area occupied by myofibrils. Each data point corresponds to the mean per animal. **e** Representative electron micrographs from each group (Scale bar: 2 μm). **f** Specific force defined as absolute (maximal) force divided by CSA, and **g** Rigor force defined as the maximum force divided by CSA in the absence of ATP (15 fibres taken from 3 animals per group). Data points correspond to individual muscle fibres in **(a–c, f, g)**. Column graphs show mean ± S.D. Scatter graphs show linear regression lines. Asterisks denote a significant difference compared with CTL (*P <  0.05; **P <  0.01; one-way ANOVA)

Optimal gene transcription and myonuclear domain sizes are thought to be a prerequisite for sufficient production of contractile proteins, the most abundant proteins in muscle fibres. Indeed, aberrant myonuclear domain sizes and altered gene transcription have been associated with reduced contractile protein content and impaired force generation in several animal models [22, 29]. We hypothesized that the same might be true in XLMTM dogs. We assessed contractile protein content via several methods. Using fluorescent labelling, the mean pixel intensity of rhodamine phalloidin (which labels actin) within individual muscle fibres was lower in the XLMTM and AAVLow animals (Fig. 6a, see also Fig. 4a). To further support this, our proteomics analysis on single muscle fibres with known CSA revealed that myosin and actin were decreased at the protein level in XLMTM when compared with results from Healthy animals (Fig. 6b, c). This is in line with our ultrastructural analyses that revealed that the XLMTM and AAVLow animals had a lower density of myofibrils within myofibres (Fig. 6d, g). AAVMid and AAVHigh treatments were capable of restoring rhodamine phalloidin staining intensity and myofibrillar density (Fig. 6a, d, e, see also Fig. 4a).

A reduction in contractile protein content might be expected to affect force generating capacity of muscle fibres. In agreement with this, specific force was significantly lower in XLMTM and AAVLow groups, compared to others. AAVMid and AAVHigh treatments restored specific force to normal levels (Fig. 6e). This force deficit could be due to changes in the total number of contractile proteins and/or myosin molecules available, their recruitment upon Ca^2+^ activation and/or their intrinsic cycling and mechanical properties in binding to actin. To distinguish between these potential mechanisms, we evaluated rigor force (maximum force divided by CSA in the absence of ATP). This parameter was significantly smaller in XLMTM and AAVLow animals than in healthy, AAVMid and AAVHigh dogs (Fig. 6f). As rigor force and specific force were decreased to a similar extent, and as under rigor conditions, all myosin heads are attached [3], we suggest that the major mechanism underlying the force depression is the availability of myosin molecules and other contractile proteins. Together, these data suggest that global nuclear synthetic activity is altered in XLMTM and AAVLow dogs versus healthy and rescued animals, and that this correlates with decreased content of contractile proteins within myofibres as well as a reduction in contractile force.

### In the absence of disease, centrally located myonuclei do not alter myofilament force generating capacity

**Fig. 7:**
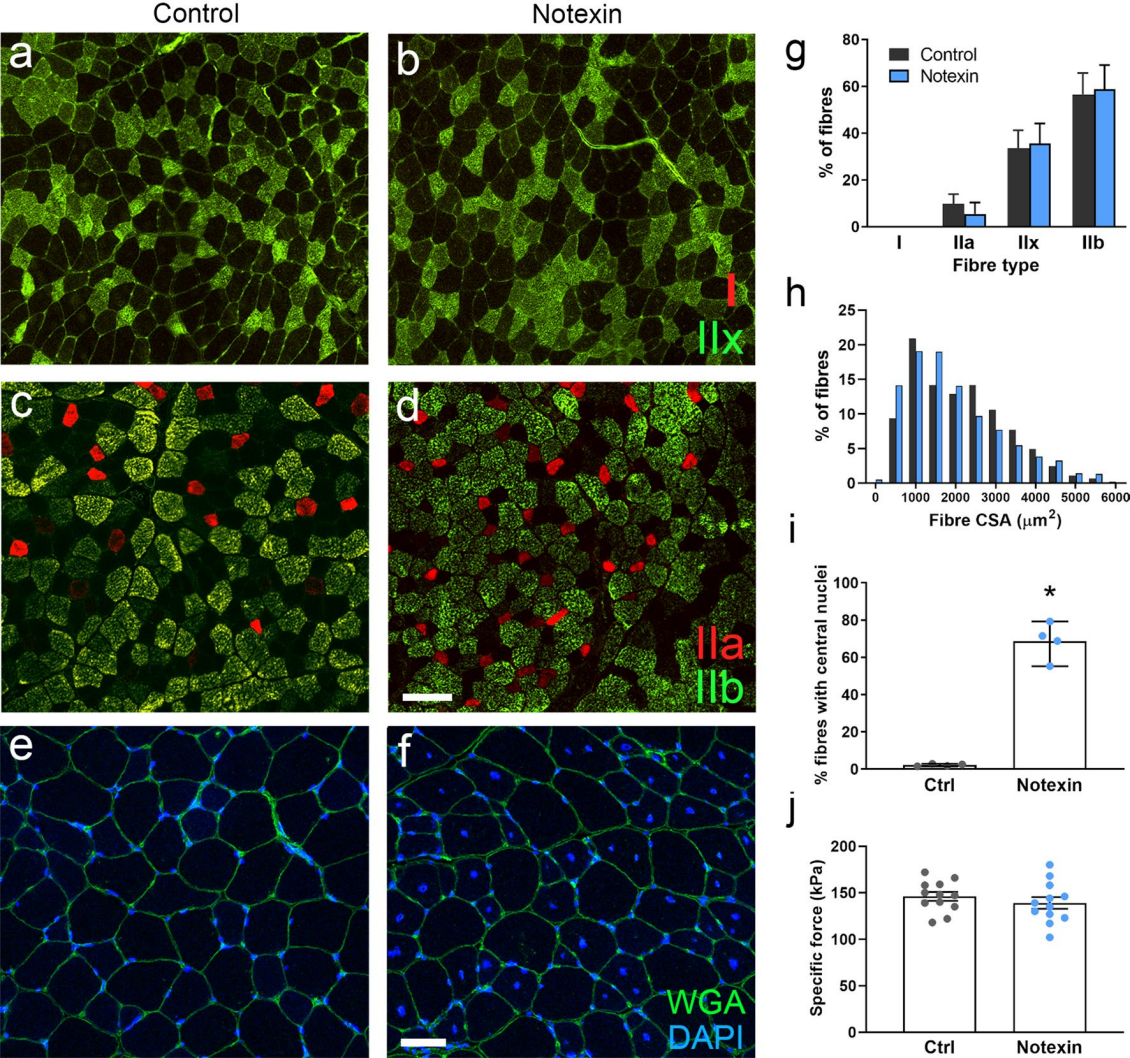
Central nucleation in the absence of disease does not affect contractile force production. Tibialis anterior muscles from wild type mice were treated with notexin to induce degeneration, followed by a period of 8 weeks for full muscle regeneration. Uninjected contralateral muscles served as controls. Muscles injured and regenerated for 8 weeks displayed similar histological parameters to the uninjured controls: proportions of type I and type II fibres **(a–d, g)**, and cross-sectional areas of fibres **(e, f, h)**. However, regenerated muscles retained a large fraction of central nuclei, absent in controls **(e, f, i)**. Despite this change, contractile force in skinned fibres was similar in both groups **(j)**. WGA (wheat-germ agglutinin), CSA, cross-sectional area. Scale bars: **a**–**d**, 50 μm; **e**, **f**, 25 μm. Individual data points in **g–i** correspond to individual animals, and in **j** to individual fibres pooled from 4 animals. Graphs show mean ± S.D. *denotes a significant difference compared to the healthy group (*P* < 0.05, *t*-test)

In addition to the abnormalities in MND size and nuclear spacing in XLMTM patients and animal models (Figs. 2–4), another form of mispositioning exists in this disease: nuclei aberrantly placed in the centre, rather than at the periphery of muscle fibres. The presence of central nuclei might have consequences for various aspects of muscle physiology, e.g. physical interference with contractile function, since central nuclei are buried among myofibrils. To determine whether this latter aspect might affect muscle fibre contraction, we used a mouse model of central nucleation, in the absence of other unrelated pathology. Tibialis anterior muscles of wild type mice were treated with notexin, resulting in muscle degeneration (N = 4 mice). Various studies have found that 3 weeks following notexin injury, muscle regeneration has occurred, resulting in the formation of new mature muscle fibres with normal histological structure, apart from the presence of centralized myonuclei (indicative of a past degeneration/regeneration event) [25]. To further exclude potential confounding effects (such as residual, low expression of developmental isoforms of contractile proteins), we allowed muscles to regenerate for 8 weeks following injury with notexin. Muscle fibres from injured legs were centrally nucleated, but they were normal in various other histological respects, resembling the uninjured, contralateral control muscles (Fig. 7a–i). These parameters included: relative expression of type I, IIa, IIb and IIx myosin heavy chain isoforms (Fig. 7a–d, g; N.B. incidences of type I fibres were < 0.2% for both groups); muscle fibre CSA (Fig. 7e, f, h), and tissue organization and architecture (Fig. 7e, f). Interestingly, the force generating capacity was similar between notexin-injured and control muscles, as assessed using skinned/permeabilized muscle fibres (Fig. 6; n = 15 fibres per condition). This suggests that the presence of central nuclei alone does not markedly interfere with muscle contractile capacity.

These results lend support to our initial hypothesis that, in the context of XLMTM, there is a novel, curable pathophysiological mechanism by which variations in nuclear mispositioning and MND volumes modify transcriptional output, which in turn affects density and/or function of the myofilaments themselves, contributing to overall weakness.

## Discussion

Here, we demonstrate that isolated muscle fibres from humans and animal models of XLMTM have an abnormally high density of myonuclei for their size (Fig. 2–4), which are irregularly spaced. In correlation with this, global synthetic capacity of these myonuclei is apparently reduced (Fig. 5), along with contractile protein content and myofilament density (Fig. 6). Consistent with these observations, force production at the myofilament level is reduced (Figs. 2, 3, 6). We also show that when *MTM1* expression is restored by systemic injection of rAAV8-cMTM1 (> 2.5 × 10^13^ vg.kg^−1^), these cellular and physiological defects are rescued in XLMTM dogs (Figs. 4–6).

### Consequences of variable myonuclear domains and activity

Other skeletal muscle diseases with altered nuclear spacing notably include those caused by mutations in genes encoding nuclear envelope proteins [21, 36], dynamin 2 (centronuclear myopathy) [12] and skeletal muscle actin/nebulin (nemaline myopathy) [31]. In light of these various disorders, it has been hypothesized that optimal muscle fibre performance requires proper myonuclear number and positioning, MND volume and synthetic activity [42]. In normal muscle fibres, myonuclear number and spacing is optimized, to minimize transport distances of gene products (i.e. mRNA transcripts) to the surrounding cytoplasmic domain. In addition, myonuclei are thought to communicate with each other, and it has been found that nuclear proteins (e.g. transcription factors) can be transported from one myonucleus to another [40]. Such a mechanism might allow inter-nuclear cooperation, where myonuclei regulate each other’s activity, thus establishing transcriptional domains within a muscle fibre. In XLMTM models, we observe an apparent reduction in global transcriptional activity (as assessed by fluorescence intensity of AcH3, a well-characterized marker of active transcription, Fig. 5), which might be related to the aberrant nuclear organization found throughout the muscle fibres. Consistent with this, we see a reduction in overall content of contractile proteins and myofilament density. Given that contractile proteins make up the vast majority of total muscle protein content, a reduction in transcriptional output might be expected to affect their abundance. Other factors that might contribute to the regulation of myofibril content in XLMTM are protein turnover systems (e.g. degradation via autophagy or proteasome), however, evidence would indicate that autophagy is partially blocked at the late stages, resulting in increased protein accumulation and aggregates, including desmin [11, 16]. This suggests a reduced protein turnover rate in XLMTM, and points towards a reduced synthesis, rather than an increased breakdown, of myofilaments in this disease.

An interesting point regarding the data on nuclear arrangement is that the large numbers of myonuclei within XLMTM muscle fibres would indicate that there is no impairment in the fusion of progenitor cells to contribute to muscle formation and growth. Rather, there is more likely a defect in the hypertrophic pathways that follow progenitor fusion. This might be in part related to abnormal transcription within nuclei, suggested in the data in Fig. 5, since growth of muscle fibres (and maintenance of size) is dependent on availability of transcripts and protein synthesis.

### Potential mechanisms of weakness in XLMTM

Various explanations for muscle weakness in XLMTM have been suggested, including reduced muscle fibre size, defective excitation–contraction coupling, impaired neurotransmission at the NMJ, and aberrant positioning of myonuclei within the centre of muscle fibres [17, 18]. However, to our knowledge, no other studies have definitively examined whether positioning of nuclei and potential downstream effects on contractility contribute to impaired muscle function in this disease. To assess this further, we measured force production in skinned/permeabilized single muscle fibres, where contraction is directly induced by applying Ca^2+^ and ATP; hence, the function of the myofilaments is examined in isolation (thus bypassing upstream events such as electrical stimulation or excitation–contraction coupling – Fig. 1). The observed reduction in specific force in muscle fibres from XLMTM patients and both animal models demonstrate that muscle function is indeed impaired at the contractile level. We propose that the primary cause of contractility defects might be the observed reduction in contractile protein/myofilament density (Fig. 6), although other more subtle factors might contribute.

Skinned single muscle fibres retain their peripheral and central nuclei (Figs. 1–4), and thus the impairments in contractility might also be caused by aberrant nuclear positioning. In particular, positioning of nuclei within the centre of muscle fibres (thus buried among the myofibrils) might be expected to interfere mechanically with contractility. To investigate this, we induced injury in the tibialis anterior of wild type mouse muscles using an intramuscular injection of notexin, followed by a period of 8 weeks for regeneration. As expected, muscles fully regenerated over this period, histologically resembling the non-injured contralateral muscles, with the exception of a large proportion of myonuclei retained at the centre of muscle fibres (Fig. 7). Again, using skinned fibres, specific force production was similar in injured and non-injured muscles, suggesting that the positioning of nuclei within the centre of the muscle fibre does not significantly interfere with muscle contractile force generation (Fig. 7). However, it is possible that more subtle effects on contractile dynamics (aside from peak force production measured here) might be affected by central nuclear positioning, for instance velocities of shortening or relaxation. In addition, other factors relating to nuclear microenvironment might be at play. The nuclear envelope is known to interact with a range of cytoskeletal components, including microtubules, desmin, and cytoplasmic/non-sarcomeric actins, all of which have roles in the distribution of mechanical forces across the cell, including in muscle fibres. Potentially, the nature of these interactions might differ in healthy versus myopathic tissue, and between peripheral and central nuclei. It is unlikely that the principal measurement here (peak force generation) is significantly affected by the arrangement of the non-sarcomeric cytoskeleton, but it might possibly have an influence on more subtle parameters of contraction, as has been found for microtubules in the modulation of contractile and relaxation velocities [20].

Another difference between the recently regenerated/notexin-treated muscles and XLMTM muscle relates to the size of muscle fibres. Diseased fibres typically have markedly reduced CSAs, whereas those treated with notexin were comparable to their control counterparts (Fig. 7f). It is possible that a central nucleus placed in a small fibre would have a greater effect than one placed in a larger fibre; hence it cannot be excluded that central nucleation in small, myopathic fibres does indeed result in an effect on muscle contractile function.

A previous study also investigated contractile properties of notexin-injured muscles, and found a small but significant reduction in specific force in mouse muscles that had been allowed to regenerate for 3 weeks, versus non-injured control muscles [15]. Our data is in agreement with this study, since we observed this phenomenon in mouse muscles regenerated for 3 weeks, but crucially, not at the longer time of 8 weeks. At 3 weeks post-notexin injection, specific forces of injured legs and non-injured tibialis anterior muscle fibres were 134.8 kPa (S.D = 15.0) and 111.5 kPa (S.D. = 17.1), respectively (*P* = 0.004, t-test, n = 15 fibres per condition from 3 mice). We propose that contractile impairments observed after short periods of regeneration might be due to incomplete muscle repair, even if histology and myosin isoform expression suggests a return to a mature state. Such differences might include a residual expression of developmental isoforms of contractile proteins other than myosin heavy chains (e.g. myosin light chains, troponins or titin splice variants [34, 43]; incomplete/immature organization of muscle architecture at the sub-histological level; or contribution of other factors. Thus, to obviate this potential issue, we assessed muscles after much longer repair periods (8 weeks), and found no difference in specific force, despite the presence of central nuclei. Additionally, regeneration events can result in branched muscle fibres, which might affect force production; however, all experiments to assess force production were carried out on small portions of individual fibres, excluding any branches.

Given that centrally positioned nuclei do not seem to have a major influence on the mechanics of contraction, it is interesting to speculate on their relevance (if any) to overall muscle function or dysfunction in XLMTM and beyond. Central positioning places the nucleus in a different environment, which might directly affect its function. It is becoming increasingly clear that mechanical cues acting on nuclei can directly regulate genome organization and transcriptional programmes, and the mechanical forces are likely to differ between centre and periphery. Interestingly, normal extraocular muscles and cardiomyocytes possess central nuclei [24], and this may be related to some as-yet unknown functional characteristic(s), or mechanical property, of these striated muscles.

**Fig. 8:**
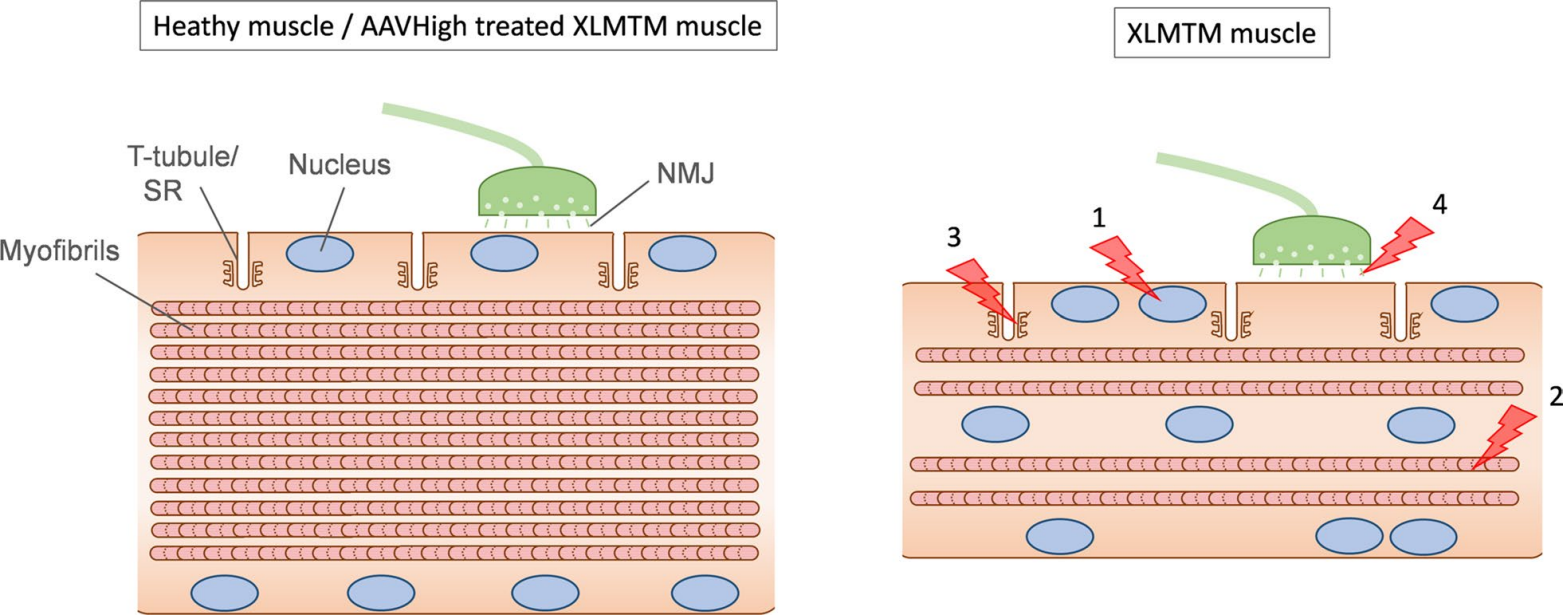
Previously known and novel pathological abnormalities in XLMTM. Muscles in animal models and patients with XLMTM display smaller muscle fibres and centrally positioned myonuclei. In addition, they display: (1) altered myonuclear organization and synthetic activity; (2) Reduced myofilament density and contractile protein content; (3) abnormal T-tubule and sarcoplasmic reticulum (SR) structure, correlated with impaired Ca^2+^ release and excitation–contraction coupling; and (4) neuromuscular junction (NMJ) and neurotransmission alterations. Other abnormalities include: altered signaling pathways; protein aggregates (e.g. desmin) potentially caused by blockade of late-stage autophagy and effects on proteasomal degradation pathways; and altered phosphoinositide metabolism (reviewed by [39])

With all these findings, as well as previous evidence, we suggest that sources of weakness in XLMTM arise from previously recognized factors such as myofibre smallness, defective excitation–contraction coupling, and NMJ alterations [17, 18], as well as a novel mechanism of impaired contractile function likely related to aberrant nuclear spacing and function, and reduced content of sarcomeric proteins/myofibrils (Fig. 8).

## Conclusion

Taken together, our data show that MND sizes, myonuclear arrangement and apparent transcriptional activity are altered in animals and patients with XLMTM. Associated with these nuclear and transcriptional defects is a reduction in contractile protein content and contractile force production. In the canine form of the disease, a single systemic dose of the *MTM1* gene can reverse these abnormalities. Muscle weakness in XLMTM is almost certainly the result of multiple factors, such as impaired excitation–contraction coupling, reduced myofibre size and NMJ alterations [17, 18]; our data indicate that abnormalities in nuclear function and contractile protein content are also likely to contribute to muscle fibre dysfunction. Lastly, our data suggests that the presence of central nuclei, which is a key feature of this disease, might not have a direct influence on muscle contractile ability, although we cannot exclude that this abnormal positioning might have other downstream effects on muscle function. This study contributes new insights into XLMTM pathology, and further highlights the benefits of gene therapy approaches in the potential treatment of this disease.
